# Supplementary material for: Factors related to the utilization of digital adherence technologies in tuberculosis care: A qualitative study among adults in DS-TB treatment, health care providers and other key actors in Tanzania
Source: PLOS Glob Public Health. 2024 Aug 8;4(8):e0002795. doi: 10.1371/journal.pgph.0002795 (PMC11309415; doi:10.1371/journal.pgph.0002795)
Supplement: Attachment — Submitted filename: Response to Reviewers_30.05.2024.docx [file pgph.0002795.s003.docx]

**Response to reviewers report on:**

**24/05/2024**

**Manuscript ID** PGPH-D-23-02462

We thank the editor and reviewer for the very valuable comments made. They have certainly enabled us to strengthen the quality of our paper and increase its relevance. In the present letter, our responses are in red. Both clean and marked-up copies of the revised manuscript are attached.

Yours Sincerely,

Bianca Tasca, corresponding author.

**Point-by-point responses**

3. Data availability statement.

R: We have added participants sociodemographic characteristics and quotes of the interviews to the manuscript. However, to protect respondents from being identified and to avoid disclosing personal and potentially stigmatizing information, the ethical review board have recommended that the underlying data not be made publicly available. Requests for access to the data can be directed to the ethical review board of Tanzania National Institute for Medical Research (NIMR).

5. Review Comments to the Author

Reviewer #1: Thank you for the opportunity to review this important research that contributes to the lack of understanding of participant and health care provider experiences using digital adherence technologies. Overall, well written and adds to the literature. The following are minor suggestions by section.

Response: We appreciate the compliments of the reviwer.

**Abstract:**
- The term ‘stakeholders’ is a common term, but it is currently recommended to use alternative terms - <https://www.cdc.gov/healthcommunication/Preferred_Terms.html>

R: Thank you for bringing the recommendations above to our attention. We have used the term “stakeholders” to refer to participants that work in Tanzania’s National Tuberculosis program and researchers engaged in Ascent. We have now taken the approach of describing their job titles when possible and to use the term “actors” as it reflects their active participation and inputs to the study.

**Introduction**
- Clear and well written

- Would be helpful to report on main findings of the trial to provide context.

R: The main trial results paper was recently published as a preprint and the trial results were now added to the manuscript. The ASCENT trial was conducted in Ethiopia, Tanzania, The Philippines, South Africa, and Ukraine. Preliminary results in Tanzania show no difference between the standard of care and people receiving DATs on the risk of poor end of treatment outcome, defined as either treatment failure, death, loss to follow-up, or a switch to a multi-drug resistant regimen (<https://ssrn.com/abstract=4720744>).

**Methods**

- Can the standard of care be more clearly defined

R: We have added more information on Tanzania’s TB standard of care (lines 136 to 140), based on Tanzania’s NTP manual for TB management, and based on observations and knowledge gained from the ASCENT study implementation.

- More information about the differential care approaches could be helpful.

R: We have added more information on the differentiated care approach based on Ascent’s protocol and guidelines (lines 120 to 123).

- Justification for inclusion of use of DAT for only one month is needed. There are likely different perspectives just starting versus using tool for longer time, e.g., 4-6 months. It might be noted in the discussion but rational for time selected is missing. And state that stage/month of use for each participant.

R: Because the study objective was to assess participants’ experiences with the DATs and factors related to their utilization, it was determined that participants should have at least one month of usage experience to provide meaningful input. We have now included a more detailed justification for the time frame selection in the manuscript (lines 159 to 161).

- Recommend using the COREQ guidelines for reporting qualitative study and report having used it to guide your report of the work. Seems that it was followed but not noted.

R: Indeed, we did utilize the COREQ guideline to assist our reporting. We have now added a reference to it in the Methods section and included the checklist as a supporting document in our submission.

- What was the rationale for not using purposive sampling to get equal numbers for each intervention type? 5 seems like a low number to get saturation for the one DAT.

R: Interviews were conducted during the Covid-19 pandemic which restricted the possibilities of purposively sampling participants. Our study population was restricted to participants that routinely visited the health unit and our initial aimed sample size was 20 participants. We believe having only 5 participants that used the medication label is a limitation of the study and have added such information to the manuscript discussion section.


- How were the smart pillboxes filled (e.g., by whom and how often)?

R: Information added in lines 111 to 112. The smart pillboxes were refilled once a month by the health care providers at the health units.

**Results**
- It might add to know the number of participants experiencing/reporting the themes to have a better sense if a theme was experienced by just one participant or the majority.

R: Given the nature of the study it is not possible to add the number of participants that concurred in a certain opinion/statement. We agree it is important to try to express if a certain aspect was restricted to a few participants or overall shared. We have added more info on that when deemed relevant.

- Results clearly written and informative. However, I was looking for more information on the actual usage of the DATs and if there were trends of use that could be corroborated with the qualitative. Maybe another paper?

R: The patterns and trends of the DATs usage has been analyzed and in fact we are planning to publish it as part of ASCENT’s process evaluation dissemination plan.

- Also, results seem lacking in patients and HCPs perception of those who stopped using the DATs or had low reporting.

R: This is another limitation of our study. Given the reasons mentioned above we were not able to recruit participants that stopped using the DATs or that were lost to follow up. This is described as part of the studies limitations (lines 537 to 541).

- Not a lot of discussion on the platform – does is show anything more than reporting calendars?

R: The adherence platform was used mainly as an adherence tracker by the HCPs. The platform was also responsible for automatically sending reminder messages to participants, which were explored in the interviews. We see this was not mentioned in the first version of the manuscript but is now included.

**Discussion**
- Do authors consider the uneven or low number of patient participants for one DAT type a potential limitation? I suggest describing either way if considered limitation or if not.

R: Thank you for bringing this up. We consider it a limitation of the study and added a description of it in the discussion section (lines 543 to 545).
